# Obstetrical Challenges in Robinow Syndrome

**DOI:** 10.1155/2022/6481517

**Published:** 2022-07-22

**Authors:** Yingao Zhang, Marco Casanova, Matthew Shanahan, V. Reid Sutton, Karin Fox

**Affiliations:** ^1^Department of Obstetrics & Gynecology, Baylor College of Medicine, Houston, TX, USA; ^2^Department of Molecular and Human Genetics, Baylor College of Medicine, Houston, TX, USA

## Abstract

Robinow syndrome is a genetically heterogenous syndrome that exhibits great pleiotropy, involving skeletal genital, cardiac, and craniofacial developmental anomalies. Fertility is not always compromised, and many individuals may be able to have a healthy pregnancy. Similar to other more common skeletal dysplasias and growth disorders such as achondroplasia, there are several challenges to be addressed in managing physiologic differences that occur in the context of pregnancy, and published literature centers on pregnant people with achondroplasia. We present a patient with Robinow syndrome (*ROR2* variant), follow her clinical course through three of her pregnancies (one 20-week loss followed by two preterm cesarean deliveries at 36-week gestation), and highlight the major obstetrical considerations in her individualized care.

## 1. Introduction

Robinow syndrome (RS) is a rare skeletal dysplasia with an estimated prevalence of 1 : 500,000 that can manifest in a constellation of different clinical presentations including mesomelia, skeletal malformations including vertebral and rib anomalies, characteristic facial features, renal and cardiac anomalies, and genital hypoplasia [1, 2]. To date, six genes with pathogenic variants (ROR2, NXN, WNT5A, FZD2, DVL1, and DVL3) have been associated with the many phenotypic presentations of Zhang et al. [3]. Genital abnormalities in affected females are generally subtle, most commonly with reduced clitoral size and hypoplasia of the labia minora, although more rare anomalies such as vaginal atresia and idiopathic hematocolpos have been described [4, 5]. Puberty takes place spontaneously with normal luteinizing hormone (LH) and follicle stimulating hormone (FSH) levels despite the increased frequency of empty sella syndrome, and there are several reports of both males and females with autosomal dominant forms of RS having children [6, 7].

Reports of RS in the obstetric literature detail the prenatal ultrasound findings in affected fetuses, as several features are detectable sonographically, including increased nuchal translucency, frontal bossing, limb shortening, digital anomalies, cleft lip and palate, and hemivertebrae [8, 9]. To our knowledge, despite reported genital hypoplasia, there are no contemporary reports that describe obstetrical care of or pregnancy risks for pregnant people with RS. Providers and patients seeking data to inform peripartum care must extrapolate from reports of pregnancy in the setting of achondroplasia. We present a case of a patient with autosomal recessive RS (ARRS) and detail her obstetric management throughout three consecutive pregnancies.

## 2. First Pregnancy

**Figure 1:**
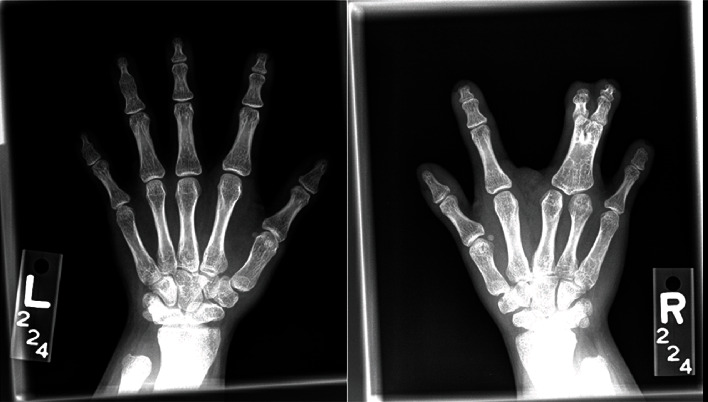
Hand XR of this patient demonstrates multiple skeletal deformities on both the left (ulnar shortening, shortened first metacarpal, hypoplastic phalanges, fusion of the middle and distal phalanges of the 4th and 5th digits) and right (ulnar shortening, shortened first metacarpal, hypoplastic phalanges, bony and soft tissue syndactyly of the 3rd and 4th proximal phalanges) hand and wrist.

**Figure 2:**
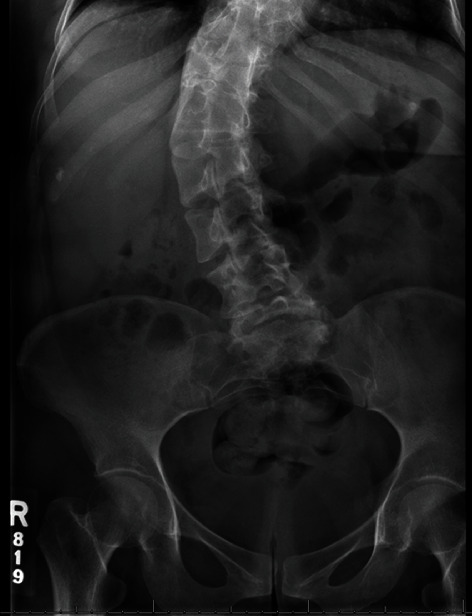
KUB and pelvic XR of this patient shows characteristic severe scoliosis often seen in RS.

A 26-year-old nulliparous Hispanic woman with clinically diagnosed Robinow syndrome presented to establish prenatal care at 10 weeks estimated gestational age. On initial physical examination, she was of mesomelic, short-limbed short stature (132.1 cm) and weight (49.8 kg). She was noted to have the following skeletal anomalies: macrocephaly (HC 59 cm, >95 percentile), brachysyndactyly (Figure 1), bilateral cleft lip and palate, broad nose, and dextroscoliosis (Figure 2). She also had a history of a congenital cardiac septal defect with spontaneous closure. Her gynecologic history was unremarkable, with reported menarche at age 11 and regular cycles lasting 3 to 4 days. She was offered and declined genetic testing at this juncture, in part due to personal preference, in part due to out-of-pocket cost.

Her pregnancy was complicated by vaginal bleeding due to a 3.5 cm subchorionic hematoma at the inferior placental margin around 17-week gestation. Though noted to be stable by serial ultrasounds, she exhibited repeated episodes of bleeding over the next two weeks. At 19 weeks, a transvaginal ultrasound found her cervical length to be shortened, measuring 15 mm. Cerclage placement was offered, which she declined and opted to start vaginal progesterone. She presented one week later with a complaint of loss of blood-tinged fluid and was found to have previable preterm premature rupture of membranes (PPROM). Painful contractions and rapid cervical changed followed, and she delivered a 315 g previable male infant vaginally. There were no dysmorphic fetal features noted following delivery, and the patient declined an autopsy and postnatal genetic testing.

## 3. Second Pregnancy

Five months after her first delivery, our patient presented to the clinic for preconception counseling. Due to her obstetrical history of subchorionic hematoma, previable PPROM with spontaneous previable delivery, she was counseled about weekly intramuscular injections of 17 alpha-hydroxyprogesterone caproate (17-OHPC) then the standard of care, as well as serial transvaginal ultrasonography to assess cervical length starting at 16-week gestation. She returned in one year for an initial prenatal visit at 8-week gestation. Once again, she was offered genetic screening and testing and declined. Her early pregnancy was uncomplicated. At 16-week gestation, transvaginal ultrasound demonstrated cervical funneling and a short cervical length of 16 mm. She began 17-OHPC injections and underwent a McDonald cervical cerclage placement. The cerclage placement was uncomplicated overall, but she was noted to have redundant, narrowed vaginal tissue at the fornices that overlapped her otherwise short, narrow cervix. Due to our patient's personal history of congenital cardiac septal defect, fetal echocardiogram was performed and was unremarkable.

At 26 weeks, mild fetal ventriculomegaly was noted on ultrasound and confirmed with follow-up MRI. The estimated fetal weight was >95th percentile, and the need for primary cesarean delivery for suspected cephalopelvic disproportion was discussed. Anesthesiology was consulted, and due to the risk of failure of neuraxial anesthesia, primary cesarean delivery under general anesthesia was planned for 37 weeks 0 days.

At 36 weeks, she presented with regular, painful contractions. Her cerclage was removed, and her cervix rapidly changed to 4 cm dilatation, with 70% effacement and -2 station. She underwent a primary low-transverse cesarean section under general anesthesia and delivered a female infant weighing 3340 grams with APGAR scores of 6 and 9 at 1 minute and 5 minutes, respectively. Her postoperative course was complicated by symptomatic anemia with Hb nadir to 6.6 g/dL, which stabilized to >10 g/dL after transfusion of 3 units of red blood cells (RBCs). The remainder of her postoperative course was uncomplicated, and she was discharged home on postoperative day 4.

## 4. Third Pregnancy

Seven months after her late preterm cesarean delivery, our patient presented for an initial prenatal visit at 7-week gestation. She opted for a history-indicated McDonald cerclage that was performed at 12 weeks and began weekly intramuscular injections of 17-OHP at 16 weeks and declined antenatal genetic testing. At 19 weeks, she began ursodiol for symptomatic, clinically diagnosed intrahepatic cholestasis of pregnancy with bile acids at 12 *μ*mol/L. The rest of her prenatal course was unremarkable, except for chronic anemia for which she took oral iron supplementation.

Given her high-risk history of previous preterm delivery, cerclage in place, and cholestasis, our patient was admitted at 36 weeks following a motor vehicle collision and underwent scheduled preterm cerclage removal, repeat cesarean section, and elective bilateral tubal ligation under general anesthesia with an estimated blood loss of 700 mL due to intractable contractions. She delivered a female infant, weighing 2885 grams, with APGAR scores of 7 and 9 and 1 minute and 5 minutes, respectively. Her postpartum course was once again complicated by symptomatic anemia with Hb nadir to 7.6 g/dL. She received two units of RBCs with appropriate rise and stabilization of her hemoglobin, and she was discharged on her third postoperative day.

**Figure 3:**
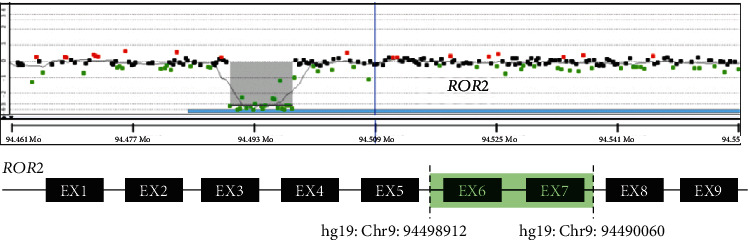
Results of aCGH for this patient revealed a homozygous deletion affecting ROR2, encompassing exons 6 and 7 confirmed by Sanger sequencing.

**Figure 4:**
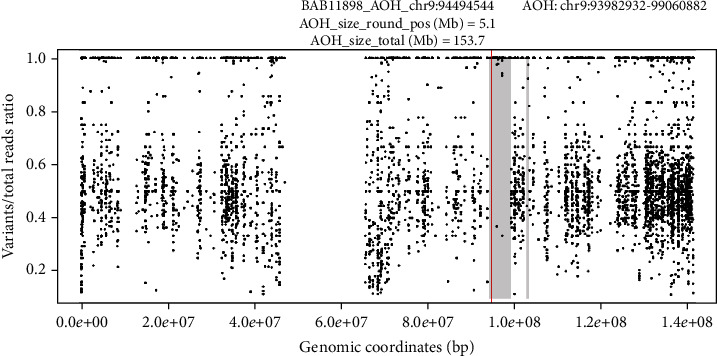
Absence of heterozygosity (AOH) plot shows the deletion within a 5.1 Mb region that suggest identity by descent (IBD) and consistent with AR inheritance.

Following her final delivery, our patient qualified for and accepted genetic testing as part of a research protocol studying genetic variations in Robinow syndrome and underwent exome sequencing and array comparative genomic hybridization (aCGH), which revealed a homozygous deletion of a ~9 kb sequence affecting exons 6 and 7 of the *ROR2* gene consistent with ARRS (Figures 3 and 4). Her partner was of normal stature, and her children did not exhibit any phenotypic features suggestive of RS. At her most recent follow-up, she was doing well two years after her final delivery.

## 5. Discussion

Although we could find no reports in the literature concerning the obstetric management of a pregnant patient with RS, there is extensive literature regarding the management of pregnant patients with more common forms of disproportionate growth disorders, such as achondroplasia and osteogenesis imperfecta [10, 11]. We took a pragmatic approach and applied published data about special considerations given to pregnant patients with achondroplasia to patients with RS, including the importance of genetic counseling, anesthesia planning, and mode of delivery concerns due to anticipated similar changes in maternal physiology, such as reduced total blood volume and body surface area common to these two conditions. However, other disease-specific risks may differ between the two conditions in pregnancy and should be considered, such as genital hypoplasia (which may not be confined to external genitalia), symptomatic anemia, and cardiopulmonary compromise [12, 13].

For patients with suspected RS, genetic counseling and testing, preferably in the preconception period, is crucial. RS is phenotypically heterogenous, and both autosomal dominant and autosomal recessive patterns of inheritance have been described [14]. Our case was confirmed to have ARRS caused by a biallelic loss-of-function homozygous deletion in *ROR2* on chromosome 9q22, which codes for the receptor-like tyrosine kinase ROR2 [15]. The deletion involving exons 6 and 7 is novel, but consistent with other reported cases of ARRS, where distinct deletion, missense, nonsense, and frameshift mutations have been reported [16]. ROR2 is involved in the noncanonical WNT-PCP signaling pathway, which is highly regulated in the differentiation of human osteoblasts during embryonic development, and variants that disrupt normal ROR2-Wnt interactions affect downstream formation and ossification of various skeletal structures [17, 18].

Due to the frequent skeletal and craniofacial abnormalities associated with RS, antenatal assessment by the anesthesia team is imperative for delivery planning. Placement of neuraxial anesthesia may be technically difficult in a patient with kyphoscoliosis or other spinal abnormalities and increase the risk of neurological complications [10]. However, general endotracheal anesthesia may also prove challenging due to the extent of specific oral, neck and craniofacial abnormalities, in addition to pregnancy-related changes of the airway ^11^. Thus, individualized planning by the anesthesiologist during the early prenatal care of the RS patient is essential to provide safe and effective intrapartum and postpartum pain management.

The mode of delivery for patients with RS should also be individualized. In achondroplasia, cesarean section is preferred due to the congenitally small and contracted pelvis in affected patients [13, 19]. However, there are reports in the literature of patients with RS with normal obstetric conjugates of the pelvis with successful vaginal deliveries [2]. This highlights the importance of assessing the adequacy of the patient's pelvis in conjunction with the estimated fetal weight and head circumference when evaluating for possible cephalopelvic disproportion and individualizing care, as illustrated from our patient's second pregnancy. This should not affect delivery timing; planned deliveries should be around 39-week gestation, and deviations should only be for other fetal or maternal indications. The decision to proceed with a preterm planned repeat cesarean section for our patient was individualized in the context of her previous preterm delivery, threatened labor, and following a motor vehicle collision. There also should be consideration of the specific genomic variant associated with RS in the patient, if genetic testing had been performed. For example, ROR2-associated RS patients tend to be of much shorter stature than patients with DVL1 mutations; there exist different phenotypic skeletal malformations with other known pathogenic variants that should be considered in the obstetrical context [3].

In contrast with patients with achondroplasia, the relative preservation of truncal height and organ sizes in those with RS may predict relatively improved obstetrical outcomes. However, the absolute decrease in thoracoabdominal volume compared to the general population may still portend to an increased risk of cardiopulmonary compromise, especially with compression from the growth of a gravid uterus [20]. This can be further exacerbated in patients with reduced baseline lung capacity secondary to severe kyphoscoliosis, as exhibited by our patient. It is essential to monitor for the development of respiratory issues with early pulmonary function testing and involvement of pulmonology colleagues if indicated. The reduced total maternal blood volume in patients with significant short stature also has implications for delivery as there may be a higher risk for symptomatic anemia after apparent low or typical volumes of blood loss, as was experienced by our patient in multiple instances. Providers must carefully monitor hemodynamic status during the 3rd and 4th stages of labor and consider a lower transfusion threshold for these patients. Drug dosing may require adjustment based on body surface area and a different volume of distribution, and close monitoring of physiologic responses to medications is recommended. Finally, this case underscores the role of genetic testing to accurately counsel patients about hereditary risk and anticipated pregnancy outcomes and the financial barriers that patients face. We hope that insurance coverage for genetic testing will be expanded as the science evolves.

## 6. Conclusion

Overall, there are multiple general clinical considerations of which to be aware regarding the obstetric management for patients with RS, a few of which have been demonstrated in the case presented above. For obstetricians, it is necessary to embrace a multidisciplinary approach for the management of these patients including not only consultation with anesthesiologists but also pulmonologists, cardiologists, neonatologists, geneticists, and radiologists. Given the diverse clinical presentation of this rare disease, an individualized approach should be emphasized to optimize both maternal and fetal outcomes.
